# Post-prandial reactive hypoglycaemia and diarrhea caused by idiopathic accelerated gastric emptying: a case report

**DOI:** 10.1186/1752-1947-5-177

**Published:** 2011-05-13

**Authors:** Stephen J Middleton, Kottekkattu Balan

**Affiliations:** 1 Department of Gastroenterology, Addenbrooke's Hospital, Cambridge University Teaching Hospital NHS Trust, Hills Road, Cambridge, CB0 2QQ, UK; 2 Department of Nuclear Medicine, Addenbrooke's Hospital, Cambridge University Teaching hospital NHS Trust, Hills Road, Cambridge, CB0 2QQ, UK

## Abstract

**Introduction:**

The majority of cases of post-prandial reactive hypoglycemia are considered idiopathic. Abnormalities of B-cell function and glucose regulation by insulin and glucagon have been postulated as causes but associated gastrointestinal dysfunction has not been reported. We report the first case of accelerated gastric emptying associated with post-prandial reactive hypoglycemia, abdominal bloating and diarrhea. We consider that gastric dysmotility is an important cause of this condition as treatment of the underlying abnormal gastric emptying allows effective control of symptoms.

**Case presentation:**

A 20-year-old Caucasian woman presented with post-prandial fatigue, sweating, nausea, faintness and intermittent confusion, which had led to pre-syncope and syncope on occasions. She also experienced marked abdominal bloating and diarrhea over the same period. These episodes responded to oral administration of sweet drinks. Her symptoms were ameliorated by modification of her diet.

**Conclusion:**

This is an original case report of the association of idiopathic accelerated gastric emptying with post-prandial reactive hypoglycemia and diarrhea. Family physicians, endocrinologists and gastroenterologists often consult patients with a constellation of post-prandial symptoms, which are considered to be idiopathic in most cases. This case indicates that gastric dysmotility might be the primary cause of these symptoms in some patients and, if found, offers a therapeutic target which in our case was successful.

## Introduction

Idiopathic post-prandial reactive hypoglycemia has been defined as a one or two hour post-prandial glucose level of ≤3.9mmol/L, or a one to two hour glucose level lower than the fasting glucose level [1]. Others have defined it as a plasma glucose level of <3mmol/L in the post-prandial period [2]. In either case the typical symptoms of hypoglycemia, such as fatigue, tremor, sweating and faintness, are required for the diagnosis and the known causes of hypoglycemia have to be excluded. Associated gastrointestinal disturbances in patients with this condition have not previously been reported, and the focus of investigations for the cause of the condition has, in the past, been on metabolic disturbances rather than gastrointestinal function. Insulin resistance and pancreatic B-cell dysfunction have been reported in a subgroup of patients with polycystic ovarian syndrome [3] whilst others have found increased sensitivity to insulin and reduced response to glucagon [4]. There remains uncertainty about the primary role of these reported abnormalities in glucose control. We report the case of a patient with post-prandial reactive hypoglycemia, diarrhea and abdominal bloating associated with idiopathic accelerated gastric emptying (IAGE), and postulate that abnormal gastric emptying may be a primary feature in some patients with these symptoms.

## Case presentation

A 20-year-old Caucasian woman presented to us after an episode of acute confusion and collapse with loss of consciousness. This was transient and she made a complete recovery without any specific treatment. She reported a two-year history of diarrhea, abdominal bloating, and nausea. She also experienced early satiety and bloating, either during or soon after eating a meal, followed by the onset of diarrhea which at worst totaled up to 15 loose stools per day. Toward the end of a diarrheal episode she often became very fatigued, shaky, sweaty, felt faint and became confused. A sweet drink resolved her symptoms.

She did not have any significant co-morbidity or family history and drank less than 10 units of alcohol per week. She did not take regular medication.

All routine blood tests and endoscopic mucosal biopsies were normal, including an HbA1c test, her thyroid status, gut hormones, a short synacthen test, and a 23-Seleno-25-homo-tauro-cholate (SeHCAT) retention study for bile salt malabsorption,.

**Figure 1:**
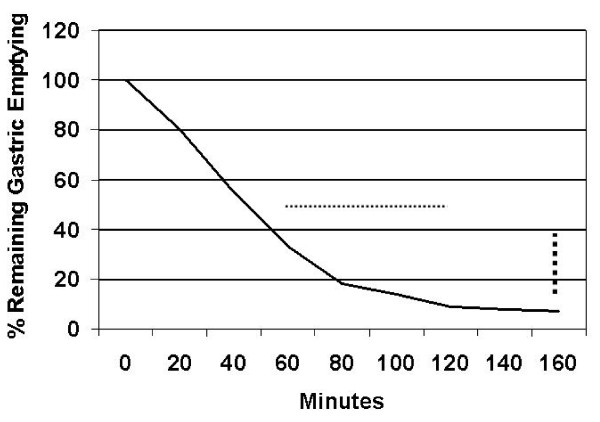
**The time for half the radio-nucleotide (99mTc-tin colloid) labeled test meal to exit the stomach (normal range given by dots) and the degree of emptying at 150 minutes (normal range small rectangles ) were reduced**.

**Figure 2:**
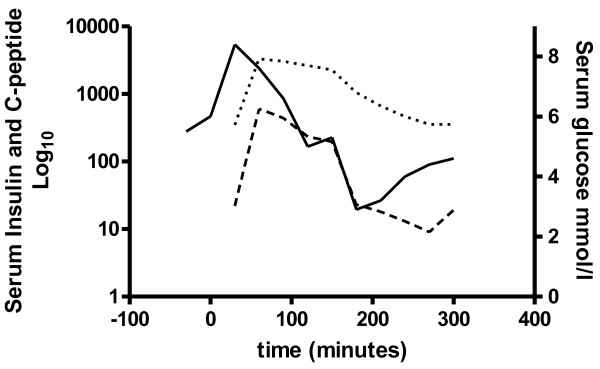
**Our patient's serum insulin (interrupted line) and C-peptide (dotted line) levels are shown in relation to serum glucose levels (continuous line) after a 50g oral glucose load taken at time zero**.

Scintigraphic measurement of gastric emptying [5] was accelerated (Figure 1). An extended glucose tolerance test was performed after a 12 hour overnight fast with a 50g oral glucose load. Her baseline fasting insulin was normal, and rose sharply after ingestion of the glucose load, remaining high at 150 minutes. Her serum glucose returned to baseline values of 5.0 and 5.3mmol/L at 125 and 150 minutes respectively and then fell to 2.9mmol/L at 180 minutes. At this point she developed symptoms consistent with hypoglycemia. Her C peptide levels were appropriate (Figure 2).

Our patient improved with dietary advice to avoid refined carbohydrates (sugars) and eat small frequent meals (a "grazing diet") rather than the usual two or three meals per day. Both her gastrointestinal and hypoglycemic symptoms continued to be well controlled with simple dietary measures at follow up 18 months later.

## Discussion

The association of IAGE with this constellation of symptoms arising from the combination of gastrointestinal disturbance and reactive hypoglycemia has not been reported previously. Similar symptoms are found in "post-gastrectomy dumping syndrome" [6] where the accelerated passage of food into the small intestine causes reactive hypoglycemia, diarrhea and bloating. We identified a similar mechanism as the likely cause of our patient's symptoms, although the cause of her accelerated gastric emptying could not be found. Severe hypoglycemia has also been reported after bariatric surgery [7,8] but has not been previously linked to IAGE. The cause of this patient's rapid gastric emptying remains uncertain. Possible causes include abnormalities in gut hormone function such as peptide YY, which is important in the control of gastric emptying and small intestinal transit [9], although this remains unclear and has not yet been investigated. An abnormality of the enteric nervous system could not be excluded because a full thickness biopsy to examine the gastric neural networks was considered too invasive to undertake in our patient.

Our patient's gastrointestinal and hypoglycemic symptoms responded well to a simple dietary strategy, which has also been used successfully in post-gastrectomy dumping syndrome. Others have reported amelioration of post-prandial hypoglycemia with acarbose, an alpha-glucosidase enzyme inhibitor [10], although its effect on associated gastrointestinal disturbance remains unknown. To the best of our knowledge, this is the first report of this condition in the literature. We consider our observations to be important as the long duration of symptoms in our patient suggests spontaneous recovery is unlikely. Patients will have long-term morbidity and frequently seek medical advice unless effective treatment is advised.

## Conclusion

This case report describes an original observation of the association of idiopathic accelerated gastric emptying with post-prandial reactive hypoglycemia and diarrhea. Reports of the syndrome of symptoms associated with this condition are relatively common in patients with functional dyspepsia and, if further investigated, a proportion of these patients may be found to have accelerated gastric emptying and thus respond to the treatment described in this case report. Family physicians, endocrinologists and gastroenterologists often consult patients with a constellation of post-prandial symptoms, which are considered to be idiopathic in most cases. This case indicates that gastric dysmotility might be the primary cause of these symptoms in some patients and, if identified, offers a therapeutic target which in our case was successful.

## Consent

Written informed consent was obtained from the patient for publication of this case report and any accompanying images. A copy of the written consent is available for review by the Editor-in-Chief of this journal.

## Competing interests

The authors declare that they have no competing interests.

## Authors' contributions

SJM undertook the clinical consultations and made the clinical observation of the association of symptoms described in this report. KB undertook the nuclear medicine investigations and interpretation of results. Both authors read and approved the final manuscript.
